# Projected Exposure of Canadian Children to Rare, High‐Severity Heatwaves Under Continued Warming

**DOI:** 10.1029/2026GH001873

**Published:** 2026-10-26

**Authors:** Masoud Zaerpour, Saeid Taleghanidoozdoozan, Brian Jacob Steele, Shadi Hatami, Lincoln Linlin Xu, Simon Michael Papalexiou, Daniel Helldén

**Affiliations:** ^1^ Department of Civil Engineering Schulich School of Engineering University of Calgary Calgary AB Canada; ^2^ Department of Geomatics Engineering Schulich School of Engineering University of Calgary Calgary AB Canada; ^3^ Centre for Health Informatics Community Health Sciences Cumming School of Medicine University of Calgary Calgary AB Canada; ^4^ Institute for Global Water Security Hamburg University of Technology Hamburg Germany; ^5^ Institutes for Transdisciplinary Scholarships University of Calgary Calgary AB Canada

**Keywords:** heatwaves, lifetime exposure, climate change projections, children’s health

## Abstract

Extreme heat is intensifying under climate change and poses disproportionate risks to children, yet Canadian children’s lifetime exposure to rare, high‐severity heatwaves remains poorly quantified at high spatial resolution. We estimate lifetime heatwave exposure across Canada under four local warming categories using bias‐corrected, downscaled CMIP6 projections aggregated to 57,932 census areas and linked with age‐structured population data. Heatwave severity is measured using the Heat Wave Magnitude Index daily (HWMId), which combines intensity and duration. A rare, high‐severity heatwave is defined as a year in which HWMId exceeds the local 98th percentile during 1981–2010—an event historically expected less than once in an average lifetime. Projected exposure increases sharply and nonlinearly with warming. Mean lifetime exposure rises from 0.44 events under 1–3°C of local warming to 4.77 under 3–5°C, 17.67 under 5–7°C, and 39.45 under 7+°C—an 89‐fold increase. The proportion of children experiencing at least one event increases from 19.8% to 84.2%, 93.7%, and 97.1% across these categories. Repeated exposure escalates even faster: no children experience five or more events under 1–3°C, compared with 39.4% under 3–5°C and 87.3% under 5–7°C. Exposure emerges earliest in western provinces, while eastern and northern regions reach similarly widespread exposure as warming increases. These findings show that historically exceptional heatwaves are projected to become recurrent over Canadian children’s lifetimes and provide spatially explicit evidence for child‐centered adaptation, including cooling infrastructure, heat‐health early warning systems, and pediatric preparedness.

## Plain Language Summary

Extreme heatwaves are becoming more frequent and intense as the climate warms, posing growing risks to children’s health. Because children are still developing physically and depend on adults and infrastructure for protection, they are especially vulnerable to heat‐related illness. This study estimates how often Canadian children may experience rare, high‐severity heatwaves over their lifetimes under different levels of warming in the places where they live. Using climate model projections combined with population data across Canada, we show that events historically rare enough to occur only once in a lifetime are likely to become increasingly common for Canadian children. Under 3–5°C of local warming, more than 80% of Canadian children are projected to experience at least one such extreme heatwave, and many will face repeated events if warming continues. Exposure is expected to emerge earliest in western Canada but will eventually affect children across nearly all regions. These findings highlight the need for stronger climate mitigation and child‐focused adaptation measures, including cooling infrastructure, early warning systems, and public health preparedness.

## Key Points

- Extreme heatwaves are projected to become common for Canadian children, with ∼84% experiencing at least one rare event under 3°C warming
- Projected lifetime exposure increases sharply with warming, with repeated high‐severity heatwave years becoming widespread under 5–7°C of local warming
- Projections across census areas reveal strong regional differences, identifying where Canadian children are likely to face the earliest and greatest heatwave exposure

## 1 Introduction

Heatwaves pose a profound threat to human health, causing intense physiological stress that can lead to acute and chronic morbidity and mortality (Campbell et al., 2018; Cavendish & Nogueira, 2025; Cheng et al., 2018; Y. Guo et al., 2017; Marchand & Gin, 2021; Romanello et al., 2025; Xu et al., 2016; Zaerpour et al., 2025). These extreme temperature events disrupt thermoregulation, precipitating conditions such as heat exhaustion, heatstroke, hyperthermia, and dehydration, often compounded by cardiovascular strain, renal failure, and electrolyte imbalances (Garg, 2025; Hajat et al., 2024). Vulnerable populations, such as older people, those with ongoing health conditions (like heart disease, breathing issues, or diabetes), and those with economic hardships, face heightened risks due to limited adaptive capacity (Kang et al., 2024). Globally, heatwaves are now responsible for tens of thousands of deaths annually, with epidemiological studies documenting sharp surges in mortality during extreme events (Alho et al., 2024; Y. Guo et al., 2017; Hopp et al., 2018). The 2003 European heatwave caused more than 70,000 deaths (Argaud et al., 2007; Johnson et al., 2005), while the 2010 Russian heatwave caused approximately 55,000 deaths, amplified by wildfires and air pollution (Shaposhnikov et al., 2014). In South Asia, recurrent heatwaves have driven increases in acute kidney injury and vector‐borne diseases (W.‐S. Lee et al., 2019; Pillai et al., 2025; Zhang et al., 2024), with a 2022 the Lancet study estimating that heat‐related deaths in India alone exceeded 20,000 annually (Garg, 2025; Hajat et al., 2024). These events not only overload emergency services but also amplify indirect health impacts, such as exacerbations of mental health disorders, including anxiety and suicidality, through sleep disruption and social isolation (Florido Ngu et al., 2021; Ramnawaz et al., 2025; R. Thompson et al., 2018).

Children are particularly susceptible to heatwave‐related harms due to their unique physiological vulnerabilities (Dang et al., 2019; Ebi et al., 2021; He et al., 2025; Schapiro et al., 2024; Sheffield et al., 2018; Xu et al., 2014). Immature thermoregulatory systems, higher surface‐area‐to‐volume ratios, and greater metabolic rates per unit body mass make children prone to rapid dehydration and hyperthermia (Azan et al., 2025; Kassem et al., 2025). Evidence links extreme heat to preterm birth, low birth weight, increased infant mortality, and a ∼16% rise in preterm birth risk during heatwaves (Chersich et al., 2020; Tian et al., 2024; Yüzen et al., 2023). Children also experience elevated rates of emergency department visits for heat‐related illnesses, asthma exacerbations, and gastrointestinal infections. Moreover, emerging evidence indicates that extreme heat also exacerbates mental health problems, including depression, and behavioral disorders (Clark et al., 2025; Proulx et al., 2024; United Nations International Children’s Emergency Fund, 2023; World Health Organization, 2021; Wright et al., 2025).

The growing frequency and severity of heatwaves under anthropogenic climate change are placing an unprecedented burden on global health systems (Alho et al., 2024; Ballarin, Oliveira, et al., 2024; Gronlund et al., 2014; Johnson et al., 2005; Romanello et al., 2024). The IPCC Sixth Assessment Report warns of longer, more intense, and more frequent events even under moderate warming scenarios (Intergovernmental Panel on Climate Change (IPCC), 2023). According to the United Nations Children’s Fund (UNICEF), nearly half of the world’s children live in areas classified as “extremely high risk” for climate impacts due to overlapping hazards such as extreme heat, drought, flood, and air pollution (Ballarin, Wendland, et al., 2024; United Nations Children’s Fund, 2024; Zaerpour et al., 2026). Recognizing this global trend, UNICEF has issued technical guidance on heat‐stress preparedness, calling for child‐centered adaptation across health, education, and infrastructure sectors (United Nations Children’s Fund, 2024).

In Canada, these global trends are intensified by accelerated warming, particularly in northern latitudes where temperatures are rising at roughly twice the global average—a result of Arctic amplification and feedbacks from melting permafrost and sea ice (Bush & Lemmen, 2019; Hatami & Nazemi, 2022; Hatami et al., 2025; Rantanen et al., 2022; Taylor et al., 2023). Projections suggest that under current policies, mean temperatures could rise 5–8°C above pre‐industrial levels by 2100, driving more frequent and severe heatwaves in the northern latitudes with serious health consequences (Davariashtiyani et al., 2023; Ford et al., 2018; Hebbern et al., 2023; Kirchmeier‐Young et al., 2025; J. Y. Lee et al., 2019; Water, Air and Climate Change Bureau, 2011). The 2021 Pacific Northwest heat dome, when temperatures reached 49.6°C in Lytton, British Columbia, caused about 619 heat‐related deaths—the deadliest weather event in Canadian history—and exposed major gaps in preparedness, including inadequate cooling infrastructure and protection (Henderson et al., 2022; Jain et al., 2024; White et al., 2023). Montreal’s 2018 heatwave similarly resulted in 66 deaths, largely among urban low‐income populations (Kenny et al., 2018; Yoon et al., 2024). Canada’s limited physiological acclimatization, low prevalence of residential air conditioning, and expanding urban heat islands further heighten vulnerability, especially in cities such as Toronto and Vancouver that still lack comprehensive heat‐resilience plans (Alberini et al., 2011; Ho et al., 2018; Kenny et al., 2018). These national examples underscore the urgent need for improved quantification of heat exposure and risk to guide evidence‐based adaptation strategies.

Despite advances in global assessments of lifetime climate‐extreme exposure, the specific impacts of heatwaves on children remain insufficiently quantified. Previous large‐scale studies have primarily assessed population‐wide or intergenerational exposure patterns using global frameworks designed to capture broad demographic trends (Grant et al., 2025; Russo et al., 2019; Thiery et al., 2021). While these efforts have advanced understanding of how climate extremes may intensify across cohorts, they inherently rely on coarse spatial resolutions and generalized demographic assumptions, for instance, static population distributions, uniform vulnerability across age groups, and simplified adaptation capacities. Such assumptions, while necessary for global comparability, may oversimplify regional climatic contrasts and social heterogeneity, thereby masking localized risks faced by vulnerable populations such as children. Children, who will live through much of the 21st century and therefore face a disproportionate share of future warming, experience heightened vulnerability. Yet few assessments disaggregate exposure by age or integrate future demographic projections, and those that do often aggregate national differences into global means, limiting their relevance for regions with strong spatial climatic contrasts, such as Canada (Bush & Lemmen, 2019). This gap highlights the need for national‐scale, demographically resolved assessments that bridge physical climate projections with population dynamics to better capture the unequal and evolving burden of heatwave exposure. Our study addresses this gap by providing the first national‐scale, child‐focused assessment of lifetime heatwave exposure in Canada, using high‐resolution climate and demographic data.

## 2 Materials and Methods

### 2.1 Data

We analyzed exposure to extreme heat across Canada using daily maximum temperature ($T_{\max}$) from two sources: observed gridded data from Daymet (Thornton et al., 2020) (Version 4; 1980–2024; 1 km) and bias‐corrected, statistically downscaled CMIP6 projections from the Canadian Downscaled Climate Scenarios (CanDCS6; 1981–2100; ∼10 km). These downscaled projections translate coarse global climate model outputs into finer regional‐scale climate information using statistical techniques that adjust model biases and better represent local climate variability, thereby providing regionally relevant and policy‐informative temperature estimates (see Supporting Information S1 for technical details). Daymet and CanDCS6 temperature fields were spatially aggregated to Statistics Canada Dissemination Areas (DAs), the smallest census geography in Canada (typically 400–700 people), producing DA‐level daily $T_{\max}$ time series for 57,932 DAs. For projections, we used 14 CMIP6 models under SSP1‐2.6, SSP2‐4.5, and SSP5‐8.5 (additional details on model selection, downscaling, and bias correction are provided in Supporting Information S1).

We obtained DA‐level child population counts from the 2021 Canadian Census, including age distributions enabling identification of children and birth cohorts. To represent future child distributions, we used provincial population forecasts and allocated projected child counts to DAs assuming the 2021 within‐province spatial age structure remained constant (Supporting Information S1). Climate and demographic data sets were aligned to a common DA geography to support consistent national‐scale exposure estimation.

### 2.2 Methodology

We quantified heatwave severity using the Heat Wave Magnitude Index daily (HWMId), an annual index that integrates heatwave intensity and duration relative to local climate conditions. In simple terms, HWMId provides a standardized measure of heatwave severity by combining the intensity and duration of heat events relative to each location’s historical climate variability, enabling comparison across regions with very different baseline climates. HWMId is a generic, population‐independent heat metric and was not modified using child‐specific physiological thresholds or vulnerability factors. The child‐specific focus of this study arises from applying the index over the childhood exposure window and linking the resulting exposure metrics with age‐structured child population data. Full methodological details are provided in Supporting Information S1. We calculated annual HWMId for each DA using Daymet (historical) and for each DA–model–scenario realization using CanDCS6 (1981–2100); full HWMId definition and parameter choices are described in Supporting Information S1.

To identify rare, high‐severity events, we defined a Lifetime‐Scale Heatwave Exposure (LHE) threshold for each DA as the 98th percentile of its annual HWMId values during the 1981–2010 baseline period. Here, “once‐in‐a‐lifetime” is used descriptively to convey rarity rather than a strict statistical return period; this threshold broadly corresponds to a multi‐decadal extreme event that would be expected only a few times over a typical lifetime. Years exceeding this DA‐specific threshold were treated as “lifetime‐scale” heatwave years for that location. Exposure metrics were calculated as counts of LHE‐exceedance years and do not incorporate the magnitude by which HWMId exceeds the threshold.

To facilitate comparison across different levels of projected climate change, each DA–model–scenario realization was grouped according to its projected local warming anomaly (ΔT), calculated relative to a historical baseline. Within these groups, local temperature anomalies (ΔT) at the DA level were analyzed to characterize regional exposure patterns. Thus, warming categories reflect warming trajectories while still capturing spatial heterogeneity in local temperature change.

To summarize results by warming level, we classified each DA–model–scenario realization using a late‐century local warming anomaly (ΔT), computed as the difference between mean annual temperature during 2071–2100 and the common 1981–2010 baseline period used to define the LHE threshold. Each realization was assigned to one of four warming bins: 1–3°C (“1°C”), 3–5°C (“3°C”), 5–7°C (“5°C”), or 7+°C (“7+°C”). Exposure outcomes were then stratified by these warming bins to enable comparison across models and scenarios while retaining local warming heterogeneity. Because warming in Canada is amplified relative to the global mean, local warming anomalies may substantially exceed corresponding global mean temperature changes. Therefore, these categories represent projected local warming at the DA level and should not be interpreted as global mean temperature levels.

To ensure clarity in terminology, we distinguish between childhood exposure and lifetime exposure throughout the study. Childhood exposure refers specifically to heatwave exposure occurring between birth and age 18, reflecting our focus on risks during early life development, whereas lifetime exposure refers more broadly to exposure accumulated over an individual’s full lifespan.

Finally, we combined DA‐level annual HWMId series, DA‐specific LHE thresholds, and child population data to estimate children’s exposure to high‐severity heatwaves. For each birth cohort and DA, we counted the number of years in which annual HWMId exceeded the LHE threshold during childhood (birth through age 18) and summarized these counts across warming bins. Population‐level estimates were obtained by weighting DA‐level exposure metrics by the number of children residing in each DA (see Supporting Information S1).

## 3 Results

Many of Canada’s most consequential heatwaves have occurred in the upper tail of local heatwave‐severity distributions—events that were historically expected to occur only rarely within a human lifetime (Figure 1).

**Figure 1:**
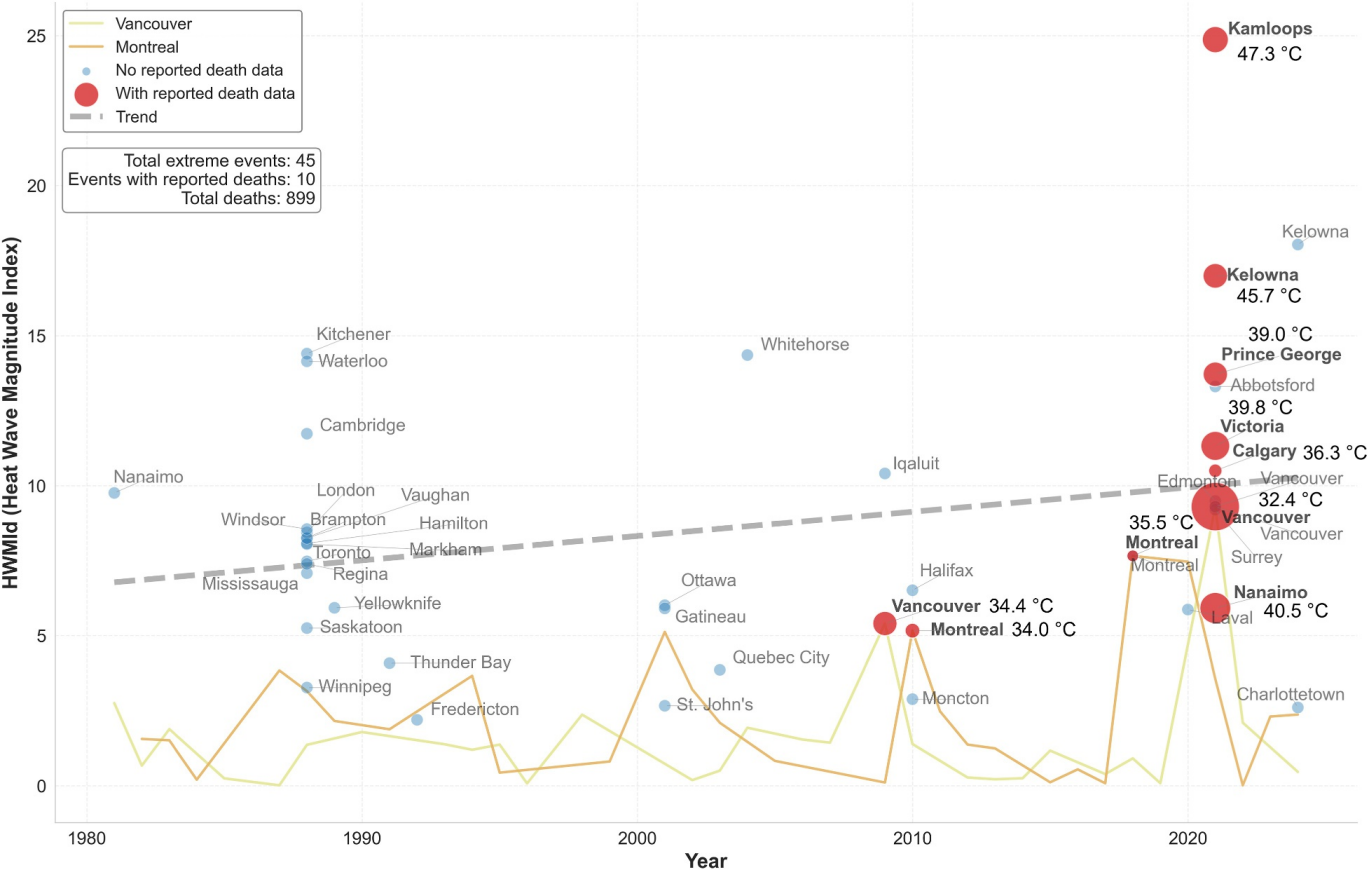
*Extreme Heatwave Events in Canadian Cities and Reported Mortality, 1981–2024*. Heatwave events exceeding the Lifetime‐Scale Heatwave Exposure (LHE) threshold are plotted by year and HWMId. This threshold represents events sufficiently rare to be statistically expected to occur less than once during an average human lifetime within the baseline climate. Identifying these LHE‐level events provides a basis for the nationwide analysis by enabling assessment of how often future generations across Canada may experience similarly exceptional heatwaves under continued warming. Bubbles are scaled by the death ratio (reported deaths divided by city population), while events without reported mortality data are shown using a fixed smaller symbol. Time series display the complete HWMId histories for the two cities with the highest reported death counts, Vancouver and Montreal, providing context for their extreme events. The maximum air temperatures associated with heatwave events for which mortality data were reported are also displayed. The dashed line represents a fitted trend across all events, illustrating the aggregate change in heatwave severity over time.

To establish the historical relevance of the Lifetime‐Scale Heatwave Exposure (LHE) threshold, we compared high‐severity heatwave events with reported heat‐related mortality across Canadian cities. Between 1981 and 2024, 45 events exceeded the city‐specific LHE threshold, of which 10 were associated with reported fatalities, accounting for 899 deaths. Approximately 70% of these deaths occurred during the most severe LHE‐level events. Across the full record, heatwave severity increased by approximately 0.81 HWMId per decade, and events associated with reported deaths had a higher mean magnitude (HWMId ≈11) than events without reported fatalities (HWMId ≈8). The 2021 heat dome in western Canada and the 2010 and 2018 Montreal heatwaves illustrate that events concentrated in the upper tail of the local HWMId distribution can have substantial mortality impacts. During the 2021 heat dome, Vancouver, Kelowna, Prince George, Victoria, and Nanaimo all exceeded their city‐specific LHE thresholds, demonstrating the broad spatial extent of this event across British Columbia. Figure 1 also presents the complete annual HWMId histories for Vancouver and Montreal, the two cities with the highest reported death counts—approximately 447 deaths in Vancouver during the 2021 heatwave and 66 deaths in Montreal during the 2018 event—together with the maximum air temperatures associated with fatal events. Together, these historical patterns support the use of LHE exceedance as a severity benchmark for evaluating future heatwave exposure across Canada.

Building on this historical evidence, we next assess how exposure to similarly severe heatwaves may accumulate during childhood in these two cities (Figure 2). Future projections show that children in both cities face rapidly accumulating exposure to LHE‐level heatwaves as warming intensifies (Figure 2). For Montreal (panel a), the cumulative number of LHE‐exceedance years (LHE threshold ≈20.6) remains negligible through 2040 under low warming trajectory, but rise sharply thereafter. By 2070 the ensemble mean reaches about 15 LHE‐exceedance years in the ≥7°C local warming category compared with 3 in the 5–7°C category and 2 in the 3–5°C category; by 2100, these counts increase to approximately 43, 18, and 5, respectively. For Vancouver, panels b–d illustrate how exposure emerges earlier and accumulates more rapidly across successive birth cohorts (local LHE threshold ≈28.0). A child born in 2030 is projected to experience about 4 LHE‐exceedance years by age 18 in the ≥7°C category, compared with approximately 1 in the 3–5°C category. For a child born in 2040, these counts rise to 8 in the ≥7°C category and 2 in the 5–7°C category. For the 2050 birth cohort, childhood exposure increases further to approximately 16 LHE‐exceedance years in the ≥7°C category, compared with 4 in the 5–7°C category and 2 in the 3–5°C category, despite the same 18‐year exposure window. Thus, for successive cohorts, events that were historically rare enough to occur only once within a lifetime become increasingly recurrent within a single childhood, with substantially greater accumulation under higher local warming conditions. These projections indicate that increasing exposure is not solely a late‐century concern but is already expected to emerge by mid‐century for children born in the coming decades.

**Figure 2:**
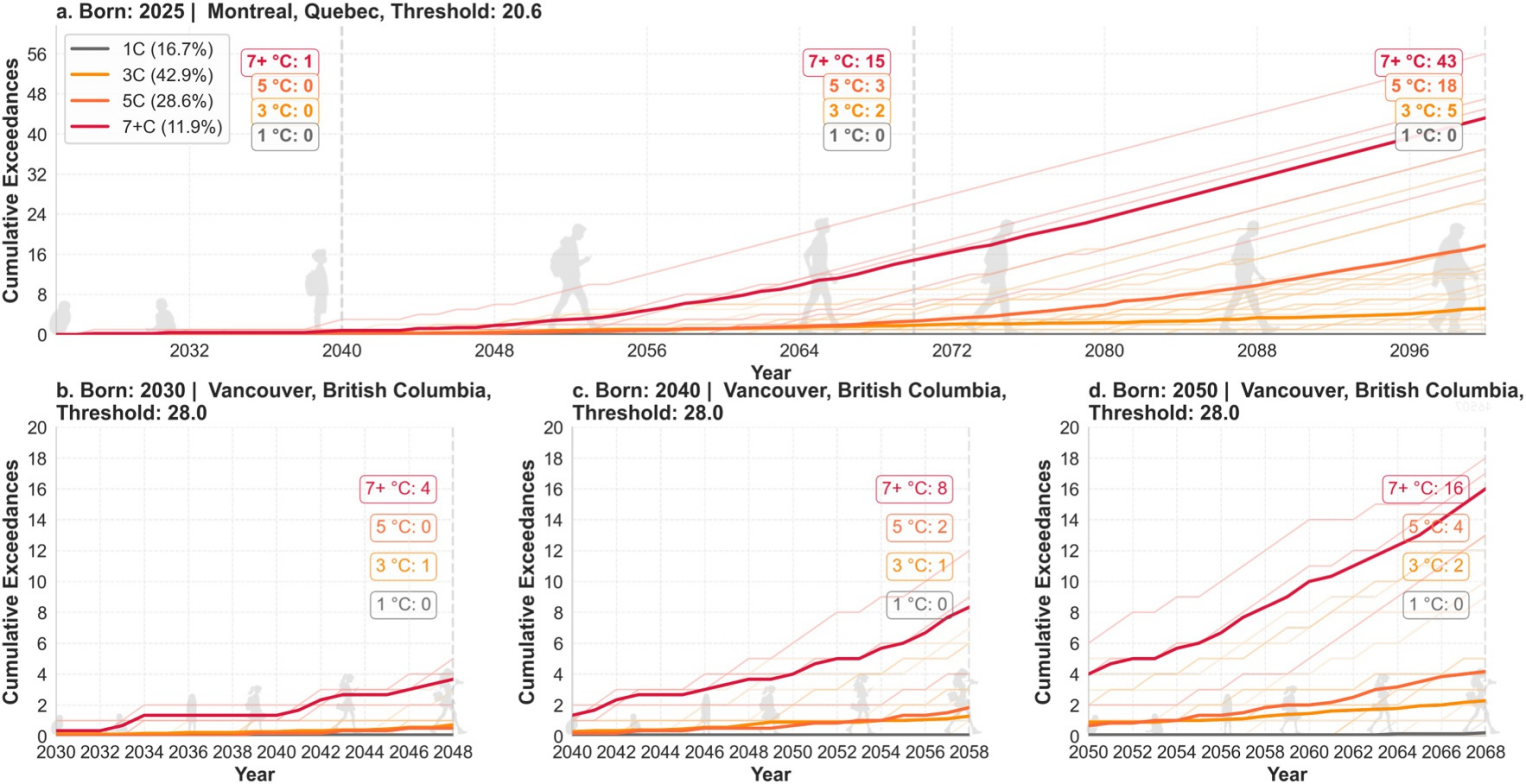
*Cumulative Heatwave Exceedance Trajectories for Montreal and Vancouver*. Projected cumulative number of years in which annual HWMId exceeds the LHE threshold across CMIP6 model realizations for Montreal and Vancouver, calculated for the period 2025–2100. Each thin line represents an individual model–scenario realization, while thick lines denote the ensemble mean for four local warming categories (1°C, 3°C, 5°C, and 7+°C). The shaded colors correspond to different warming levels, with darker tones representing higher global temperature trajectories. Panel (a) illustrates exceedance accumulation for Montreal from 2025 to 2100, including vertical dashed lines marking the 2040, 2070, and 2100 time horizons. Panels (b–d) depict projected cumulative counts of exceedances during the childhood periods of individuals born in 2030, 2040, and 2050 in Vancouver, respectively, extending to age 18 (2048, 2058, and 2068). Colored boxes in each panel summarize the expected number of extreme heatwave events by the end of each period under different warming trajectories.

Vancouver (panels b–d) show how this escalation emerges earlier into childhood for successive birth cohorts (local threshold ≈28.0). A child born in 2030 is projected to encounter about 4 LHE exceedances by age 18 under 7+°C (and ∼1 under 3°C); for a child born in 2040, those counts rise to 8 (7+°C) and 2 (5°C); by 2050, childhood exposure approximately doubles again to 16 (7+°C) with 4 under 5°C and 2 under 3°C, despite the same age window. Thus, for successive cohorts, events that were once “once‐in‐a‐lifetime” become increasingly routine within a single childhood, with steeper accumulation under higher global‐warming pathways. These cities were chosen because they have already suffered the deadliest heatwaves in Canada (Vancouver, ∼447 deaths during the 2021 heatwave; Montreal, ∼66 during the 2018 event), and the projections show that exceedance growth is not only a late‐century problem but is underway by mid‐century for cohorts now entering school.

We extend the analysis over the Dissemination Area (DA) level across the whole country. At DA level, Figure 3 reveals a distinct spatial and temporal evolution of heatwave exceedances across Canada, transitioning from sparse, low‐frequency events in the near future to widespread and persistent exposure by the end of the century. Spatially, the maps show how small, localized areas of elevated exceedance under 1°C, mostly confined to southern and coastal regions, expand into continuous, high‐exposure zones across much of the country as warming intensifies. Temporally, the hexagon summaries highlight how this intensification unfolds through three distinct phases: near‐term (2025–2040), mid‐century (2041–2070), and end‐of‐century (2071–2100), with each successive period showing clear upward shifts in provincial averages.

**Figure 3:**
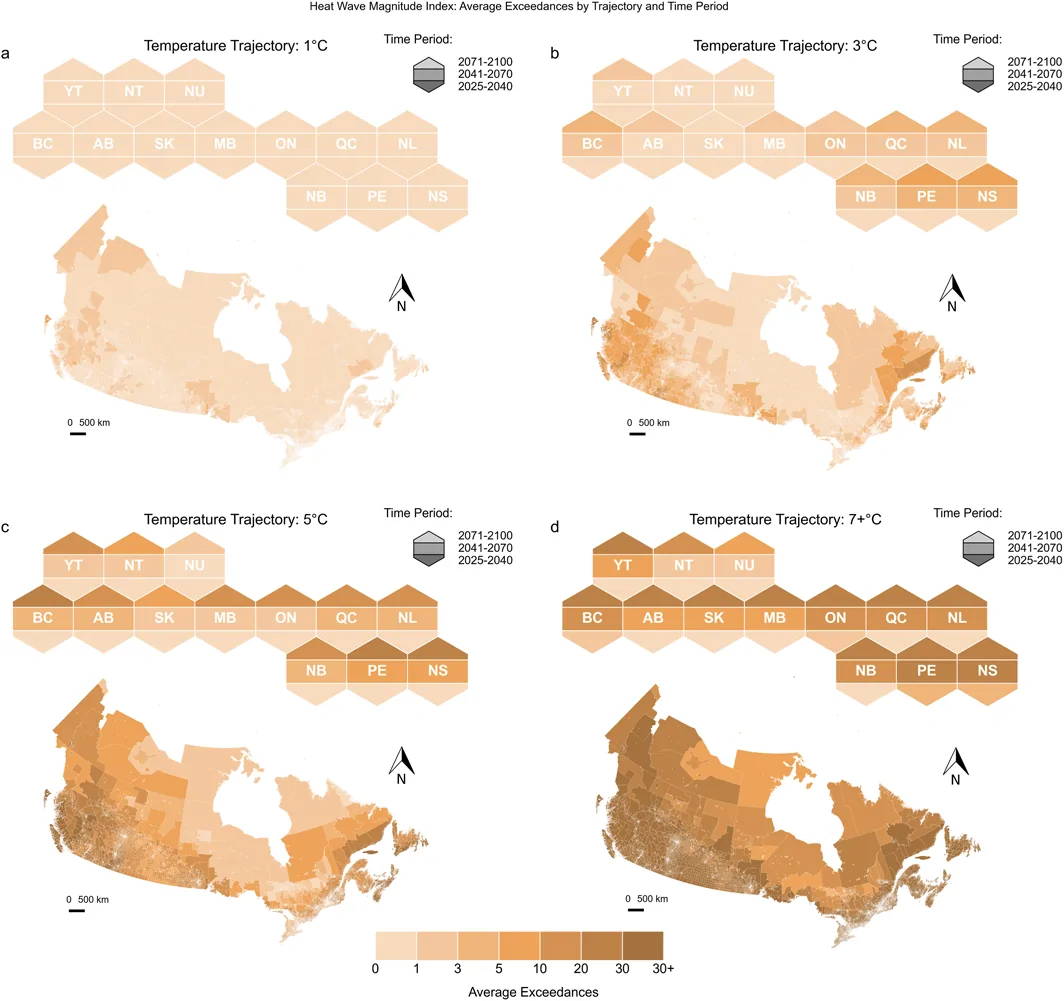
*Heatwave Magnitude Index (HWMId): Nationwide Summaries and DA‐Level Maps by Warming Trajectory and Time Period*. Cumulative counts of LHE‐exceedance years based on DA‐specific thresholds are shown for Canada under four local warming trajectories (1°C, 3°C, 5°C, 7+°C). In each panel, the hexagon row summarizes province‐level means, with each province split into three horizons that represent near‐term (2025–2040), mid‐century (2041–2070), and end‐of‐century (2071–2100) averages. The map displays the corresponding Dissemination‐Area (DA) distribution across Canada. Color indicates number of times that LHE threshold is exceeded. Panel (a) (1°C) highlights limited exceedance accumulation nationally, with most provinces and DAs remaining in the lowest bins across all periods. Panel (b) (3°C) shows a clear intensification, particularly by mid‐century and end‐of‐century in western, central and Atlantic provinces. Panel (c) (5°C) indicates widespread increases in provincial means and DA‐level exceedances, with large contiguous regions shifting into higher bins. Panel (d) (7+°C) exhibits the strongest escalation, with most provinces showing dark bands at end‐of‐century and extensive high‐exceedance areas on the DA maps, underscoring the rapid growth in extreme‐heat exposure under high‐warming pathways.

During the near‐term (2025–2040), exceedances remain limited in magnitude and spatial extent. National means stay below 1 event, and most provinces still fall within the lowest exposure ranges, indicating that extreme heat events will still occur sporadically rather than systematically. By mid‐century (2041–2070), however, a visible transformation emerges. Under 3°C and higher trajectories, provincial averages increase severalfold, reaching ∼1.6 exceedances nationally at 3°C and 2.9 at 5°C, with expanded areas of moderate exceedance stretching from coastal British Columbia to the Atlantic provinces. This period marks the onset of recurring high‐magnitude heatwaves at a national scale, signaling the emergence of sustained, recurring conditions that transform extreme heat events from isolated anomalies into a defining feature of the new climatic norm. The end‐of‐century (2071–2100) projections show the most dramatic acceleration. Under 5°C, national averages rise to 14.5 exceedances, and under 7+°C they reach 27.1, implying that many DAs will experience several extreme heat events within the same generation. The top hexagon bands in Prince Edward Island, Nova Scotia, and British Columbia reach the uppermost exposure levels, reflecting gains of over +20 to +28 exceedances between early‐ and late‐century periods. By 2100, nearly all provinces surpass the ≥10‐event threshold, and most exceed ≥20, confirming that extreme heat will become a consistent feature rather than an anomaly. Overall, the figure reveals a clear temporal escalation: limited exceedance in the near term, rapid expansion by mid‐century, and dominance of extreme‐exceedance conditions across most regions by 2100, underscoring the steady, decade‐by‐decade intensification.

Building on the national‐scale intensification of heatwave risk shown in Figure 3, we next examine how this evolving hazard translates into lifetime exposure for children. As the only cohort that will experience the full arc of 21st‐century warming, children face uniquely prolonged and cumulative risk. Figure 4 shows that children in Canada are projected to face far greater and more frequent exposure to LHE‐level heatwave as the climate warms, but this escalation will unfold unevenly across the country, with regional disparities shaped by climate, proximity to coasts, and elevation. Nationwide averages of cumulative exceedances rise nonlinearly from 0.44 events under 1°C to 4.77 at 3°C, 17.67 at 5°C, and 39.45 under 7+°C, representing an almost 89‐fold increase in lifetime exposure. Translating these exceedances into population impacts, the share of children reaching at least one LHE event climbs from 19.8% at 1°C to 84.2% at 3°C, 93.7% at 5°C, and 97.1% at 7+°C. For more intense thresholds, the escalation is even steeper: No children exceed ≥5 LHEs at 1°C, but this rises to 39.4% at 3°C, 87.3% at 5°C, and 96.7% at 7+°C—and by 7+°C, more than 80% of Canadian children experience ≥20 LHE events in their lifetimes.

**Figure 4:**
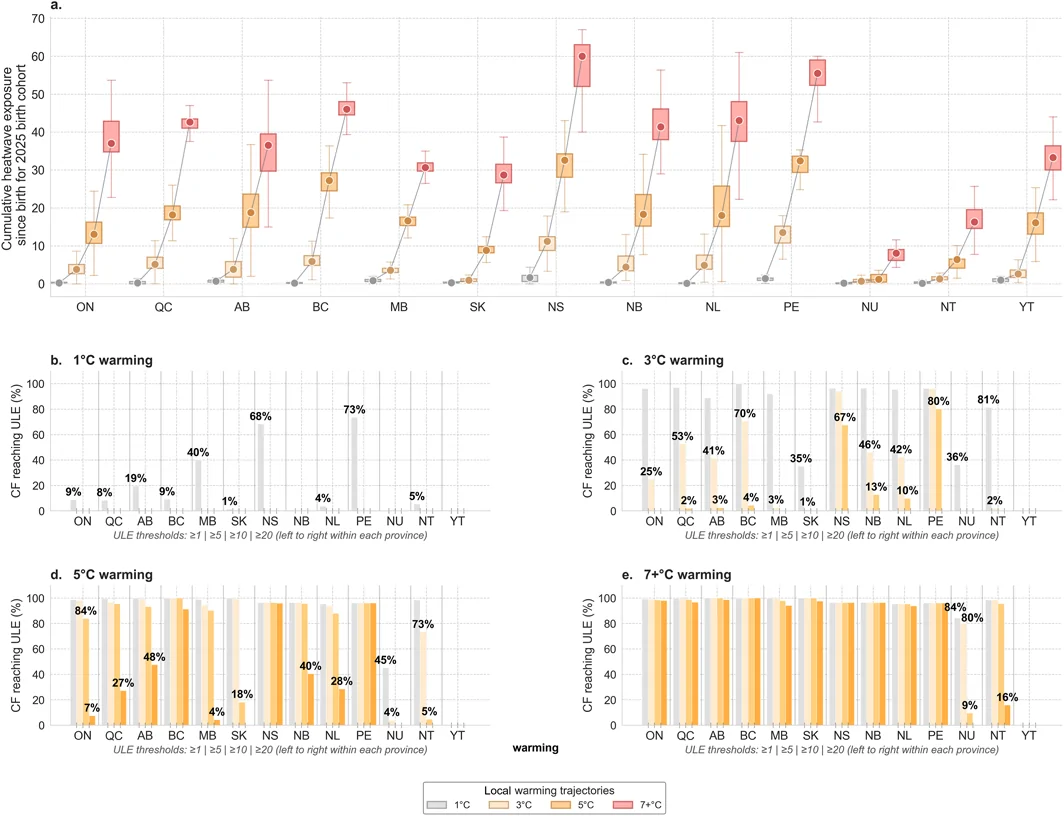
*Provincial Pattern of Children*’*s Heatwave Exposure in Canada under Warming Trajectories (2025 Birth Cohort)*. Nationwide assessment of children’s cumulative heatwave exposure at the Dissemination Area (DA) level, summarized by province for the 2025 birth cohort. Exposure is based on DA‐level cumulative counts of years in which annual HWMId exceeds the DA‐specific LHE threshold and summarized under four local warming trajectories (1°C, 3°C, 5°C, 7+°C). Provinces are ordered (left→right) by number of children in census 2021. Colors represent warming trajectories consistently across panels. Panel (a) Province‐wise distributions of DA average cumulative heatwave exposure since birth for each warming trajectory. Boxes show interquartile ranges. The gray lines highlights shift with warming across trajectories. Panel (b) (1°C), Panel (c) (3°C), Panel (d) (5°C), Panel (e) (7+°C) display share of children (%) in each province whose cumulative exposure reaches at least the LHE thresholds of ≥1, ≥5, ≥10, and ≥20 events (left to right within each province) for each warming trajectory. Value labels are shown for bars ≤90% to aid comparison without clutter.

This intensification, however, is geographically uneven. Children in the western provinces, British Columbia, Alberta, and Saskatchewan, reach full exposure earliest: by 5°C, nearly 100% of children encounter at least one LHE event, and saturation persists for higher thresholds (≥10 and ≥20 LHEs) under 7+°C. In contrast, the eastern provinces, such as Prince Edward Island and Nova Scotia, begin with comparatively higher exposure even under modest warming (73.5% and 68.2% of children above the ≥1 LHE threshold at 1°C), reflecting maritime heat risks. By 5–7+°C, both east and west converge toward full LHE exposure, with children in Nova Scotia and PEI exceeding 55 mean events, among the highest nationwide. Children in the northern territories including Yukon, Nunavut, and Northwest Territories will experience relatively lower exposure but still pronounced increases compared with current climates.

## 4 Discussion

The projections presented in this study indicate a dramatic escalation in Canadian children’s exposure to extreme heat, with far‐reaching implications for child health (Romanello et al., 2021, 2024; Tian et al., 2024; Wright et al., 2025; Zhang et al., 2024). Under a moderate warming scenario of ∼3°C by 2100, approximately 84% of today’s Canadian children are expected to experience at least one LHE‐level heatwave in their lifetime, whereas under a high‐end ∼5°C scenario this share rises to 94%. Our DA‐level analysis further shows that lifetime exposure counts rise nonlinearly, from 0.44 LHE events under 1°C to 4.77 at 3°C, 17.67 at 5°C, and 39.45 under 7+°C, an almost 89‐fold increase in severe heatwave years over the course of a child’s life. Perhaps more worrying, nearly 9 in 10 children under the 5°C trajectory would endure five or more such once‐rare events, signaling that recurrent exposure will become the norm rather than the exception for the coming generation. At the highest warming level, more than 80% of Canadian children are projected to experience at least 20 LHE‐level events, implying repeated exposure over multiple developmental stages. In effect, what were historically “once‐in‐a‐lifetime” heat extremes are on track to become predictable, population‐wide hazards for the next generation (Grant et al., 2025; Romanello et al., 2021; Russo et al., 2019; Thiery et al., 2021). The implications for child health are profound (Li et al., 2025; Sheffield et al., 2018; Tsuzuki, 2023; Vergunst & Berry, 2022; World Health Organization, 2021; Wright et al., 2025) and underscore that safeguarding children’s health in a warming world will require both aggressive climate mitigation and child‐focused adaptation measures.

The burden on Canada’s health system is also set to increase substantially, with important regional disparities in timing and scale of impact (Boudreault et al., 2025; X. Guo et al., 2024; Romanello et al., 2024). Western provinces (British Columbia, Alberta, and Saskatchewan) are projected to reach near‐universal child exposure to LHE heatwaves earliest (Azan et al., 2025; Grant et al., 2025). This reflects the fact that these regions are already experiencing intense heat anomalies; for instance, the 2021 Pacific Northwest “heat dome” brought Lytton, BC to 49.6°C and resulted in approximately 619 excess heat‐related deaths in British Columbia alone (Henderson et al., 2022; V. Thompson et al., 2022; White et al., 2023; Yoon et al., 2024). Such events, occurring decades before 2100, indicate the acute burden on healthcare: during the 2021 heat emergency, ambulance services and hospitals in BC were overwhelmed, revealing systemic unpreparedness (e.g., a lack of cooling in homes and long‐term care facilities) (Baumbusch et al., 2025; Henderson et al., 2022; Korfmacher et al., 2025). Our projections indicate that the western provinces will face these challenges first and most frequently. However, by mid‐century even historically cooler regions in central and eastern Canada, as well as the sub‐Arctic north, rapidly catch up in exposure rates (Rantanen et al., 2022). Any initial maritime or latitudinal buffer erodes as warming intensifies, meaning no part of the country is insulated from extreme heat by the 2050s. The 2018 Quebec heatwave, which led to 66 deaths in the Montreal area, tragically demonstrated that eastern Canada is also vulnerable when extreme heat strikes urban populations without adequate preparedness (Yoon et al., 2024; Zou et al., 2026). Together, these events highlight how unprecedented heat has been putting high pressure on Canada’s health infrastructure. This emerging crisis calls for urgent enhancements in heat‐health preparedness: provincial health authorities will need robust heat action plans, early warning systems, expanded surge capacity for emergency services, and safeguards for critical infrastructure such as power grids and cooling centers to prevent failures during concurrent heat and health emergencies (Romanello et al., 2024).

Notably, our findings align with and extend global analyses of climate‐exposure inequity, reinforcing that today’s children face an unprecedented climate burden (Gauffin & Spencer, 2022; Helldén et al., 2021; Parsons et al., 2025). Worldwide, children born now are expected to experience many times more extreme events than their grandparents. Projections suggest that children born in 2020 will face several times more heatwaves than those born in 1960, with 50%–90% of the cohort exposed to extreme lifetime heat events by 2100 depending on the warming pathway (Grant et al., 2025; Russo et al., 2019; Thiery et al., 2021). Our Canada‐specific, high‐resolution mapping at the Dissemination‐Area level, where the rate of climate warming is roughly twice the global average, reveals that this pattern is not only replicated but intensified. Essentially, an entire generation of Canadian youth will be exposed to extremes that exceed the range of historical climate variability. Moreover, we find that a large share will encounter multiple such events: if warming reaches ∼5°C, nearly 87% of Canadian children are projected to experience five or more LHE‐level heatwaves, a cumulative exposure burden that exceeds global mean projections. This compounding of risk is especially ominous for health. Without substantial interventions, a future of recurrent high‐magnitude heatwaves could produce a dramatic rise in heat‐related morbidity and mortality among Canada’s population. In this context, our study adds quantitative evidence from a high‐income, northern nation to the global narrative of climate injustice: children, who have contributed least to climate change, will bear a vastly greater health burden from its effects (Gauffin & Spencer, 2022; Parsons et al., 2025).

Beyond aggregate statistics, this research contributes a granular perspective on climate risk by leveraging high‐resolution data and a child‐centric lens (Brunner & Voigt, 2024; Rajulapati et al., 2022; Rubin et al., 2025; Yu et al., 2025). We integrated downscaled CMIP6 projections with the HWMId to identify LHE‐level heatwaves and mapped these risks at the DA scale across Canada. Our approach, informed by census‐based population distributions and provincial demographic forecasts, provides insight into which communities and sub‐populations are most at risk, and when. It builds on the global frameworks by adding age‐specific and location‐specific granularity. For instance, the analysis can distinguish how a child growing up in a dense urban neighborhood might face an earlier and higher number of extreme heat exposures compared to a child in a coastal or rural area, due to differences in microclimate and adaptive capacity (Hsu et al., 2021; Lin et al., 2025; Yang et al., 2024). Such fine‐scale resolution is crucial for evidence‐based adaptation: it enables policymakers to target interventions (e.g., cooling centers, greening initiatives, health services) to the districts that will need them most. In essence, our findings move beyond national averages to highlight the uneven geographic distribution of heat risk—indicating that extreme heat emergence will be nationwide in extent but heterogeneous in timing and intensity, requiring geographically tailored adaptation measures (Mohamed et al., 2025).

Beyond direct heat exposure, these findings also have broader planetary‐health implications. The warming that drives more frequent and severe heatwaves can simultaneously disrupt the ecosystems on which human health and well‐being depend, particularly in Arctic and sub‐Arctic regions (Constable et al., 2022; Schipper et al., 2022). Changes in snow and ice conditions, permafrost, freshwater systems, species distributions, and ecological seasonality may affect food and water security, access to the land, land‐based livelihoods, and cultural continuity in northern communities. Although these ecosystem responses were not directly evaluated in this study, the findings indicate that heat adaptation should not be considered solely as a public‐health or infrastructure challenge. Policy responses should integrate child‐focused health protection with ecosystem conservation, climate‐resilient land‐use and infrastructure planning, and community‐led approaches that incorporate Indigenous knowledge and local priorities.

Some limitations should be acknowledged. First, this study quantifies exposure to high‐severity heatwaves rather than direct health outcomes. The reported LHE exceedance counts and population fractions indicate the frequency and distribution of exposure to extreme heat but should not be interpreted as direct estimates of heat‐related morbidity, mortality, or healthcare burden. Although substantial evidence links extreme heat exposure to adverse health outcomes in children, translating exposure into quantitative health impacts would require the application of exposure–response relationships together with information on population vulnerability, adaptation capacity, and healthcare access. Future work could integrate epidemiological models with the exposure framework developed here to estimate potential health burdens under future warming scenarios. A second limitation relates to demographic assumptions. Future population exposure was estimated using projected provincial population growth while maintaining the 2021 within‐province age structure and spatial distribution of children. Consequently, the analysis does not account for future demographic shifts, migration, urbanization, or changes in age composition that could alter the geographic distribution of exposure.

## 5 Conclusion

Taken together, our study shows that extreme heat will become a predictable, population‐wide hazard for the coming generation. The rapid growth in LHE exceedances across all warming trajectories highlights that mitigation remains critical: limiting warming to 1°C avoids tens of extreme events per child compared to 3–7+°C futures. At the same time, adaptation strategies must center children, whose physiology, dependence, and cumulative exposure make them uniquely vulnerable to long‐term health consequences. Strengthening Canada’s pediatric climate preparedness across health systems, schools, housing, emergency management, and social services will be essential to safeguarding child health in a warmer future. These measures should form part of broader ecosystem‐informed and community‐led adaptation strategies, particularly in northern regions where health, livelihoods, food security, and cultural continuity are closely connected to rapidly changing natural systems.

## Conflict of Interest

The authors declare no conflicts of interest relevant to this study.

## Supporting information

Supporting Information S1

## Data Availability

This study used three publicly accessible data sets. Downscaled future climate projections were obtained from the Canadian Downscaled Climate Scenarios (CanDCS6) archive developed by Environment and Climate Change Canada, which provides bias‐adjusted CMIP6 daily temperature projections at approximately 10 km resolution using the BCCAQv2 and MBCn downscaling methods (Cannon, 2018; Cannon et al., 2015; Werner & Cannon, 2016). Historical daily maximum temperature fields were sourced from Daymet Version 4, a 1‐km–resolution gridded data set covering North America and hosted by the Oak Ridge National Laboratory Distributed Active Archive Center (Thornton et al., 2020, Rule 1). Population and demographic data at the Dissemination Area level were obtained from the 2021 Canadian Census, through Statistics Canada’s Census Program (Government of Canada, 2021). All data sets are publicly available and were harmonized within a common geospatial framework to support the DA‐level climate and population analyses conducted in this study. The Python notebooks and supporting replication files used to reproduce the calculations and main manuscript figures are archived on Zenodo (Zaerpour, 2026).

## References

[gh270219-bib-0001] Alberini, A. , Gans, W. , & Alhassan, M. (2011). Individual and public‐program adaptation: Coping with heat waves in five cities in Canada. International Journal of Environmental Research and Public Health, 8(12), 4679–4701. 10.3390/ijerph8124679 PMC3290981 22408596

[gh270219-bib-0002] Alho, A. M. , Oliveira, A. P. , Viegas, S. , & Nogueira, P. (2024). Effect of heatwaves on daily hospital admissions in Portugal, 2000–18: An observational study. The Lancet Planetary Health, 8(5), e318–e326. 10.1016/S2542-5196(24)00046-9 38729671

[gh270219-bib-0003] Argaud, L. , Ferry, T. , Le, Q.‐H. , Marfisi, A. , Ciorba, D. , Achache, P. , et al. (2007). Short‐ and long‐term outcomes of heatstroke following the 2003 heat wave in Lyon, France. Archives of Internal Medicine, 167(20), 2177–2183. 10.1001/archinte.167.20.ioi70147 17698677

[gh270219-bib-0004] Azan, A. , Nyimbili, S. , Babayode, O. O. , & Bershteyn, A. (2025). Exceeding the limits of paediatric heat stress tolerance: The risk of losing a generation to climate inaction. BMJ Paediatrics Open, 9(1), e002883. 10.1136/bmjpo-2024-002883 PMC11784133 39842863

[gh270219-bib-0005] Ballarin, A. S. , Oliveira, P. T. S. , Uchôa, J. G. S. M. , Lima, C. H. R. , Zaerpour, M. , Godoy, M. R. V. , et al. (2024). Escalating threat of human‐perceived heatwaves in Brazil. Environmental Research Communications, 6(11), 111004. 10.1088/2515-7620/ad9140

[gh270219-bib-0006] Ballarin, A. S. , Wendland, E. , Zaerpour, M. , Hatami, S. , Meira Neto, A. A. , & Papalexiou, S. M. (2024). Frequency rather than intensity drives projected changes of rainfall events in Brazil. Earth’s Future, 12(1), e2023EF004053. 10.1029/2023EF004053

[gh270219-bib-0007] Baumbusch, J. , Fong, V. , Lee, E. , Bandara, N. A. , & Khan, K. B. (2025). Weathering the storm: Climate‐related weather event experiences of families of children with medical complexity. Child, 51(2), e70068. 10.1111/cch.70068 PMC11919613 40102035

[gh270219-bib-0008] Boudreault, J. , Campagna, C. , Lavigne, É. , & Chebana, F. (2025). Projecting the overall heat‐related health burden and associated economic costs in a climate change context in Quebec, Canada. Science of the Total Environment, 958, 178022. 10.1016/j.scitotenv.2024.178022 39674157

[gh270219-bib-0009] Brunner, L. , & Voigt, A. (2024). Pitfalls in diagnosing temperature extremes. Nature Communications, 15(1), 2087. 10.1038/s41467-024-46349-x PMC10948863 38499558

[gh270219-bib-0010] Bush, E. , & Lemmen, D. S. (2019). Canada’s changing climate report (p. 444) [News releases]. Government of Canada. Retrieved from https://changingclimate.ca/site/assets/uploads/sites/2/2020/06/CCCR_FULLREPORT‐EN‐FINAL.pdf

[gh270219-bib-0011] Campbell, S. , Remenyi, T. A. , White, C. J. , & Johnston, F. H. (2018). Heatwave and health impact research: A global review. Health & Place, 53, 210–218. 10.1016/j.healthplace.2018.08.017 30189362

[gh270219-bib-0012] Cannon, A. J. (2018). Multivariate quantile mapping bias correction: An N‐dimensional probability density function transform for climate model simulations of multiple variables. Climate Dynamics, 50(1), 31–49. 10.1007/s00382-017-3580-6

[gh270219-bib-0013] Cannon, A. J. , Sobie, S. R. , & Murdock, T. Q. (2015). Bias correction of GCM precipitation by quantile mapping: How well do methods preserve changes in quantiles and extremes? 10.1175/JCLI-D-14-00754.1

[gh270219-bib-0014] Cavendish, T. A. , & Nogueira, T. (2025). A call to action: Climate leadership at the World Health Assembly. The Lancet Planetary Health, 101389. 10.1016/j.lanplh.2025.101389 41290016

[gh270219-bib-0015] Cheng, J. , Xu, Z. , Bambrick, H. , Su, H. , Tong, S. , & Hu, W. (2018). Heatwave and elderly mortality: An evaluation of death burden and health costs considering short‐term mortality displacement. Environment International, 115, 334–342. 10.1016/j.envint.2018.03.041 29627746

[gh270219-bib-0016] Chersich, M. F. , Pham, M. D. , Areal, A. , Haghighi, M. M. , Manyuchi, A. , Swift, C. P. , et al. (2020). Associations between high temperatures in pregnancy and risk of preterm birth, low birth weight, and stillbirths: Systematic review and meta‐analysis. BMJ, 371, m3811. 10.1136/bmj.m3811 PMC7610201 33148618

[gh270219-bib-0017] Clark, D. G. , Liang, K. E. , Cheng, I. , Ford, J. D. , & Gossack‐Keenan, K. (2025). Climate disaster effects on acute health care: A case study and model of the 2021 heatwave in British Columbia, Canada. The Lancet Planetary Health, 9(5), e356–e363. 10.1016/S2542-5196(25)00075-0 40381630

[gh270219-bib-0018] Constable, A. J. , Harper, S. , Dawson, J. , Holsman, K. , Mustonen, T. , Piepenburg, D. , & Rost, B. (2022). Cross‐chapter paper 6: Polar regions. In Climate change 2022: Impacts, adaptation and vulnerability. Cambridge University Press. 10.1017/9781009325844.023

[gh270219-bib-0019] Dang, T. N. , Honda, Y. , Van Do, D. , Pham, A. L. T. , Chu, C. , Huang, C. , & Phung, D. (2019). Effects of extreme temperatures on mortality and hospitalization in Ho Chi Minh City, Vietnam. International Journal of Environmental Research and Public Health, 16(3), 432. 10.3390/ijerph16030432 PMC6388260 30717328

[gh270219-bib-0020] Davariashtiyani, A. , Taherkhani, M. , Fattahpour, S. , & Vitousek, S. (2023). Exponential increases in high‐temperature extremes in North America. Scientific Reports, 13(1), 19177. 10.1038/s41598-023-41347-3 PMC10628173 37932278

[gh270219-bib-0021] Ebi, K. L. , Capon, A. , Berry, P. , Broderick, C. , de Dear, R. , Havenith, G. , et al. (2021). Hot weather and heat extremes: Health risks. The Lancet, 398(10301), 698–708. 10.1016/S0140-6736(21)01208-3 34419205

[gh270219-bib-0022] Florido Ngu, F. , Kelman, I. , Chambers, J. , & Ayeb‐Karlsson, S. (2021). Correlating heatwaves and relative humidity with suicide (fatal intentional self‐harm). Scientific Reports, 11(1), 22175. 10.1038/s41598-021-01448-3 PMC8593067 34782650

[gh270219-bib-0023] Ford, J. D. , Couture, N. , Bell, T. , & Clark, D. G. (2018). Climate change and Canada’s north coast: Research trends, progress, and future directions. Environmental Reviews, 26(1), 82–92. 10.1139/er-2017-0027

[gh270219-bib-0024] Garg, R. (2025). Rising heat, failing kidneys: Heat stress nephropathy in climate crisis. RMC Global Journal, 1(2), 48–57. 10.25259/RMCGJ_17_2025

[gh270219-bib-0025] Gauffin, K. , & Spencer, N. (2022). Climate crisis and child health inequity. BMJ Paediatrics Open, 6(1), e001357. 10.1136/bmjpo-2021-001357 PMC8984059 36053643

[gh270219-bib-0026] Government of Canada, S. C. (2021). 2021 census of population—Data products. https://www12.statcan.gc.ca/census‐recensement/2021/dp‐pd/index‐eng.cfm

[gh270219-bib-0027] Grant, L. , Vanderkelen, I. , Gudmundsson, L. , Fischer, E. , Seneviratne, S. I. , & Thiery, W. (2025). Global emergence of unprecedented lifetime exposure to climate extremes. Nature, 641(8062), 374–379. 10.1038/s41586-025-08907-1 PMC12058528 40335711

[gh270219-bib-0028] Gronlund, C. J. , Zanobetti, A. , Schwartz, J. D. , Wellenius, G. A. , & O’Neill, M. S. (2014). Heat, heat waves, and hospital admissions among the elderly in the United States, 1992–2006. Environmental Health Perspectives, 122(11), 1187–1192. 10.1289/ehp.1206132 PMC4216145 24905551

[gh270219-bib-0029] Guo, X. , Weinberger, K. R. , Tamburic, L. , Peters, C. E. , & McLeod, C. B. (2024). Heat‐related illness among workers in British Columbia, Canada: Extreme hot weather in 2021 compared to 2001–2020. Scandinavian Journal of Work, Environment & Health, 50(7), 545–554. 10.5271/sjweh.4179 PMC11474959 39083050

[gh270219-bib-0030] Guo, Y. , Gasparrini, A. , Armstrong, B. G. , Tawatsupa, B. , Tobias, A. , Lavigne, E. , et al. (2017). Heat wave and mortality: A multicountry, multicommunity study. Environmental Health Perspectives, 125(8), 087006. 10.1289/EHP1026 PMC5783630 28886602

[gh270219-bib-0031] Hajat, S. , Casula, A. , Murage, P. , Omoyeni, D. , Gray, T. , Plummer, Z. , et al. (2024). Ambient heat and acute kidney injury: Case‐crossover analysis of 1 354 675 automated e‐alert episodes linked to high‐resolution climate data. The Lancet Planetary Health, 8(3), e156–e162. 10.1016/S2542-5196(24)00008-1 38453381

[gh270219-bib-0032] Hatami, S. , & Nazemi, A. (2022). Compound changes in temperature and snow depth lead to asymmetric and nonlinear responses in landscape freeze–thaw. Scientific Reports, 12(1), 2196. 10.1038/s41598-022-06320-6 PMC8828744 35140288

[gh270219-bib-0033] Hatami, S. , Zaerpour, M. , Ballarin, A. S. , Adamowski, J. F. , Papalexiou, S. M. , Pietroniro, A. , & Kimball, J. S. (2025). Declining number of northern hemisphere land‐surface frozen days under global warming and thinner snowpacks. Communications Earth & Environment, 7(1), 29. 10.1038/s43247-025-03059-6

[gh270219-bib-0034] He, C. , Zhu, Y. , Bachwenkizi, J. , Chen, R. , Kan, H. , & Fawzi, W. W. (2025). Assessment of heatwave impacts on child feeding practices across 36 low‐income and middle‐income countries: A cross‐sectional analysis. The Lancet Planetary Health, 9(8), 101281. 10.1016/j.lanplh.2025.06.005 PMC12447361 40712629

[gh270219-bib-0035] Hebbern, C. , Gosselin, P. , Chen, K. , Chen, H. , Cakmak, S. , MacDonald, M. , et al. (2023). Future temperature‐related excess mortality under climate change and population aging scenarios in Canada. Canadian Journal of Public Health = Revue Canadienne de Santé Publique, 114(5), 726–736. 10.17269/s41997-023-00782-5 PMC10484859 37308698

[gh270219-bib-0036] Helldén, D. , Andersson, C. , Nilsson, M. , Ebi, K. L. , Friberg, P. , & Alfvén, T. (2021). Climate change and child health: A scoping review and an expanded conceptual framework. The Lancet Planetary Health, 5(3), e164–e175. 10.1016/S2542-5196(20)30274-6 33713617

[gh270219-bib-0037] Henderson, S. B. , McLean, K. E. , Lee, M. J. , & Kosatsky, T. (2022). Analysis of community deaths during the catastrophic 2021 heat dome. Environmental Epidemiology, 6(1), e189. 10.1097/EE9.0000000000000189 PMC8835552 35169667

[gh270219-bib-0038] Ho, H. C. , Knudby, A. , Chi, G. , Aminipouri, M. , & Yuk‐FoLai, D. (2018). Spatiotemporal analysis of regional socio‐economic vulnerability change associated with heat risks in Canada. Applied Geography, 95, 61–70. 10.1016/j.apgeog.2018.04.015 PMC6482004 31031454

[gh270219-bib-0039] Hopp, S. , Dominici, F. , & Bobb, J. F. (2018). Medical diagnoses of heat wave‐related hospital admissions in older adults. Preventive Medicine, 110, 81–85. 10.1016/j.ypmed.2018.02.001 PMC6040588 29428173

[gh270219-bib-0040] Hsu, A. , Sheriff, G. , Chakraborty, T. , & Manya, D. (2021). Disproportionate exposure to urban heat island intensity across major US cities. Nature Communications, 12(1), 2721. 10.1038/s41467-021-22799-5 PMC8149665 34035248

[gh270219-bib-0041] Intergovernmental Panel on Climate Change (IPCC) . (2023). Weather and climate extreme events in a changing climate. In Climate change 2021—The physical science basis: Working group I contribution to the sixth assessment report of the intergovernmental panel on climate change (pp. 1513–1766). Cambridge University Press. 10.1017/9781009157896.013

[gh270219-bib-0042] Jain, P. , Sharma, A. R. , Acuna, D. C. , Abatzoglou, J. T. , & Flannigan, M. (2024). Record‐breaking fire weather in North America in 2021 was initiated by the Pacific northwest heat dome. Communications Earth & Environment, 5(1), 202. 10.1038/s43247-024-01346-2

[gh270219-bib-0043] Johnson, H. , Kovats, R. S. , McGregor, G. , Stedman, J. , Gibbs, M. , Walton, H. , et al. (2005). The impact of the 2003 heat wave on mortality and hospital admissions in England. Health Statistics Quarterly, 25, 6–11. 15804164

[gh270219-bib-0044] Kang, Y. , Park, J. , & Jang, D.‐H. (2024). Compound impact of heatwaves on vulnerable groups considering age, income, and disability. Scientific Reports, 14(1), 24732. 10.1038/s41598-024-75224-4 PMC11494128 39433792

[gh270219-bib-0045] Kassem, H. , Lavigne, E. , Weinberger, K. , & Brauer, M. (2025). Extreme heat and pediatric health in a warming world: A space‐time stratified case‐crossover investigation in Ontario, Canada. Environmental Health, 24(1), 35. 10.1186/s12940-025-01153-y PMC12145649 40483440

[gh270219-bib-0046] Kenny, G. P. , Flouris, A. D. , Yagouti, A. , & Notley, S. R. (2018). Towards establishing evidence‐based guidelines on maximum indoor temperatures during hot weather in temperate continental climates. Temperature: Multidisciplinary Biomedical Journal, 6(1), 11–36. 10.1080/23328940.2018.1456257 PMC6422495 30906809

[gh270219-bib-0047] Kirchmeier‐Young, M. C. , Li, G. , Wan, H. , & Zhang, X. (2025). Heat wave trends in Canadian regions. Atmosphere‐Ocean, 63(4), 241–251. 10.1080/07055900.2025.2521501

[gh270219-bib-0048] Korfmacher, M. , Hartwell, C. , Hill, K. , Matthews‐Trigg, N. , Hess, J. , Nori‐Sarma, A. , et al. (2025). Lessons learnt from the 2021 Pacific Northwest heat dome: A qualitative study of western Washington’s healthcare community response. BMJ Open, 15(4), e089093. 10.1136/bmjopen-2024-089093 PMC12007061 40246560

[gh270219-bib-0049] Lee, J. Y. , Kim, H. , Gasparrini, A. , Armstrong, B. , Bell, M. L. , Sera, F. , et al. (2019). Predicted temperature‐increase‐induced global health burden and its regional variability. Environment International, 131, 105027. 10.1016/j.envint.2019.105027 31351381

[gh270219-bib-0050] Lee, W.‐S. , Kim, W.‐S. , Lim, Y.‐H. , & Hong, Y.‐C. (2019). High temperatures and kidney disease morbidity: A systematic review and meta‐analysis. Journal of Preventive Medicine and Public Health, 52(1), 1–13. 10.3961/jpmph.18.149 PMC6378387 30742756

[gh270219-bib-0051] Li, P. , Wu, J. , Xue, T. , & Zhu, T. (2025). Associations between monthly temperature anomalies and mortality among children younger than five years in low‐ and middle‐income countries. Communications Medicine, 5(1), 365. 10.1038/s43856-025-01101-4 PMC12370998 40841773

[gh270219-bib-0052] Lin, J. , Yang, G. , Nie, W. , Wang, Z. , Chen, H. , Pan, X. , et al. (2025). Disparities in heat exposure between urban and rural populations: A case study of the Yangtze River Delta. Sustainable Cities and Society, 131, 106774. 10.1016/j.scs.2025.106774

[gh270219-bib-0053] Marchand, M. , & Gin, K. (2021). The cardiovascular system in heat stroke. CJC Open, 4(2), 158–163. 10.1016/j.cjco.2021.10.002 PMC8843991 35198932

[gh270219-bib-0054] Mohamed, M. , Amin, S. , Lever, E. , Montini, A. , Machida, K. , Rajagopalan, S. , et al. (2025). Climate change and child wellbeing: A systematic evidence and gap map on impacts, mitigation, and adaptation. The Lancet Planetary Health, 9(4), e337–e346. 10.1016/S2542-5196(25)00061-0 40252680

[gh270219-bib-0055] Parsons, E. S. , Jowell, A. , Veidis, E. , Barry, M. , & Israni, S. T. (2025). Climate change and inequality. Pediatric Research, 98(4), 1238–1245. 10.1038/s41390-024-03153-z PMC12549322 38914758

[gh270219-bib-0056] Pillai, A. , Murage, P. , & Haque, F. (2025). Understanding the relationship between climate change‐related meteorological factors and chronic kidney disease (CKD) and CKD of unknown origin (CKDu) in low‐ and middle‐income countries: A systematic review & meta‐analysis. Environmental Research, 286, 122928. 10.1016/j.envres.2025.122928 40992449

[gh270219-bib-0057] Proulx, K. , Daelmans, B. , Baltag, V. , & Banati, P. (2024). Climate change impacts on child and adolescent health and well‐being: A narrative review. Journal of Global Health, 14, 04061. 10.7189/jogh.14.04061 PMC11115477 38781568

[gh270219-bib-0058] Rajulapati, C. R. , Gaddam, R. K. , Nerantzaki, S. D. , Papalexiou, S. M. , Cannon, A. J. , & Clark, M. P. (2022). Exacerbated heat in large Canadian cities. Urban Climate, 42, 101097. 10.1016/j.uclim.2022.101097

[gh270219-bib-0059] Ramnawaz, S. , Lavigne, E. , Augustinavicius, J. , & Weichenthal, S. (2025). Short‐term variations in summer temperatures and mental health‐related emergency department visits in Ontario, Canada. Environmental Epidemiology, 9(6), e431. 10.1097/EE9.0000000000000431 PMC12548982 41140315

[gh270219-bib-0060] Rantanen, M. , Karpechko, A. Y. , Lipponen, A. , Nordling, K. , Hyvärinen, O. , Ruosteenoja, K. , et al. (2022). The Arctic has warmed nearly four times faster than the globe since 1979. Communications Earth & Environment, 3(1), 168. 10.1038/s43247-022-00498-3

[gh270219-bib-0061] Romanello, M. , McGushin, A. , Napoli, C. D. , Drummond, P. , Hughes, N. , Jamart, L. , et al. (2021). The 2021 report of the Lancet Countdown on health and climate change: Code red for a healthy future. The Lancet, 398(10311), 1619–1662. 10.1016/S0140-6736(21)01787-6 PMC7616807 34687662

[gh270219-bib-0062] Romanello, M. , Walawender, M. , Hsu, S.‐C. , Moskeland, A. , Palmeiro‐Silva, Y. , Scamman, D. , et al. (2024). The 2024 report of the Lancet Countdown on health and climate change: Facing record‐breaking threats from delayed action. The Lancet, 404(10465), 1847–1896. 10.1016/S0140-6736(24)01822-1 PMC7616816 39488222

[gh270219-bib-0063] Romanello, M. , Walawender, M. , Hsu, S.‐C. , Moskeland, A. , Palmeiro‐Silva, Y. , Scamman, D. , et al. (2025). The 2025 report of the Lancet Countdown on health and climate change. The Lancet. 10.1016/S0140-6736(25)01919-1

[gh270219-bib-0064] Rubin, H. J. , Zhang, L. , Fu, J. S. , Rastogi, D. , Kao, S.‐C. , & Ashfaq, M. (2025). Dynamically downscaled seasonal heat wave projections in the CONUS. npj Climate and Atmospheric Science, 8(1), 233. 10.1038/s41612-025-01055-3

[gh270219-bib-0065] Russo, S. , Sillmann, J. , Sippel, S. , Barcikowska, M. J. , Ghisetti, C. , Smid, M. , & O’Neill, B. (2019). Half a degree and rapid socioeconomic development matter for heatwave risk. Nature Communications, 10(1), 136. 10.1038/s41467-018-08070-4 PMC6329840 30635557

[gh270219-bib-0066] Schapiro, L. H. , McShane, M. A. , Marwah, H. K. , Callaghan, M. E. , & Neudecker, M. L. (2024). Impact of extreme heat and heatwaves on children’s health: A scoping review. The Journal of Climate Change and Health, 19, 100335. 10.1016/j.joclim.2024.100335 PMC12851172 41647315

[gh270219-bib-0067] Schipper, E. L. F. , Revi, A. , Preston, B. L. , Carr, E. R. , Eriksen, S. H. , Fernandez‐Carril, L. R. , et al. (2022). Climate resilient development pathways. In Climate change 2022: Impacts, adaptation and vulnerability. Cambridge University Press. 10.1017/9781009325844.027

[gh270219-bib-0068] Shaposhnikov, D. , Revich, B. , Bellander, T. , Bedada, G. B. , Bottai, M. , Kharkova, T. , et al. (2014). Mortality related to air pollution with the Moscow heat wave and wildfire of 2010. Epidemiology, 25(3), 359–364. 10.1097/EDE.0000000000000090 PMC3984022 24598414

[gh270219-bib-0069] Sheffield, P. E. , Herrera, M. T. , Kinnee, E. J. , & Clougherty, J. E. (2018). Not so little differences: Variation in hot weather risk to young children in New York City. Public Health, Special Issue on Health and High Temperatures, 161, 119–126. 10.1016/j.puhe.2018.06.004 PMC6633908 29960726

[gh270219-bib-0070] Taylor, P. C. , Langen, P. L. , & Tan, I. (2023). Editorial: Arctic amplification: Feedback process interactions and contributions. Frontiers in Earth Science, 11, 1140871. 10.3389/feart.2023.1140871

[gh270219-bib-0071] Thiery, W. , Lange, S. , Rogelj, J. , Schleussner, C.‐F. , Gudmundsson, L. , Seneviratne, S. I. , et al. (2021). Intergenerational inequities in exposure to climate extremes. Science, 374(6564), 158–160. 10.1126/science.abi7339 34565177

[gh270219-bib-0072] Thompson, R. , Hornigold, R. , Page, L. , & Waite, T. (2018). Associations between high ambient temperatures and heat waves with mental health outcomes: A systematic review. Public Health, 161, 171–191. 10.1016/j.puhe.2018.06.008 30007545

[gh270219-bib-0073] Thompson, V. , Kennedy‐Asser, A. T. , Vosper, E. , Lo, Y. T. E. , Huntingford, C. , Andrews, O. , et al. (2022). The 2021 western North America heat wave among the most extreme events ever recorded globally. Science Advances, 8(18), eabm6860. 10.1126/sciadv.abm6860 PMC9067932 35507648

[gh270219-bib-0074] Thornton, M. M. , Shrestha, R. , Wei, Y. , Thornton, P. E. , & Kao, S.‐C. (2020). Daymet: Daily surface weather data on a 1‐km grid for North America, version 4. ORNL DAAC. 10.3334/ORNLDAAC/1840

[gh270219-bib-0075] Tian, T. , Liang, B. , Zhang, Y. , Huang, T. , Shi, C. , Wang, P. , et al. (2024). Extreme heat and heatwaves are linked to the risk of unintentional child injuries in Guangzhou city. Communications Earth & Environment, 5(1), 665. 10.1038/s43247-024-01846-1

[gh270219-bib-0076] Tsuzuki, K. (2023). Effects of heat exposure on the thermoregulatory responses of young children. Journal of Thermal Biology, 113, 103507. 10.1016/j.jtherbio.2023.103507 37055125

[gh270219-bib-0077] United Nations Children’s Fund . (2024). A threat to progress: Confronting the effects of climate change on child health and well‐being. United Nations.

[gh270219-bib-0078] United Nations International Children’s Emergency Fund . (2023). Protecting children from heat stress (A technical note). United Nations International Children’s Emergency Fund. Retrieved from https://www.unicef.org/media/139926/file/Protecting‐children‐from‐heat‐stress‐A‐technical‐note‐2023.pdf

[gh270219-bib-0079] Vergunst, F. , & Berry, H. L. (2022). Climate change and children’s mental health: A developmental perspective. Clinical Psychological Science, 10(4), 767–785. 10.1177/21677026211040787 PMC9280699 35846172

[gh270219-bib-0080] Water, Air and Climate Change Bureau . (2011). Extreme heat events guidelines, technical guide for health care workers. Health Canada.

[gh270219-bib-0081] Werner, A. T. , & Cannon, A. J. (2016). Hydrologic extremes—An intercomparison of multiple gridded statistical downscaling methods. Hydrology and Earth System Sciences, 20(4), 1483–1508. 10.5194/hess-20-1483-2016

[gh270219-bib-0082] White, R. H. , Anderson, S. , Booth, J. F. , Braich, G. , Draeger, C. , Fei, C. , et al. (2023). The unprecedented Pacific Northwest heatwave of June 2021. Nature Communications, 14(1), 727. 10.1038/s41467-023-36289-3 PMC9910268 36759624

[gh270219-bib-0083] World Health Organization . (2021). Investing in our future: A comprehensive agenda for the health and well‐being of children and adolescents. World Health Organization. Retrieved from https://www.who.int/publications/i/item/9789240037793

[gh270219-bib-0084] Wright, C. Y. , Norval, M. , Naidoo, N. , Bulani, M. , Coovadia, A. , & Theron, L. (2025). The impact of rising ambient temperatures on the mental and physical health of children. Hygiene and Environmental Health Advances, 15, 100137. 10.1016/j.heha.2025.100137

[gh270219-bib-0085] Xu, Z. , FitzGerald, G. , Guo, Y. , Jalaludin, B. , & Tong, S. (2016). Impact of heatwave on mortality under different heatwave definitions: A systematic review and meta‐analysis. Environment International, 89(90), 193–203. 10.1016/j.envint.2016.02.007 26878285

[gh270219-bib-0086] Xu, Z. , Sheffield, P. E. , Su, H. , Wang, X. , Bi, Y. , & Tong, S. (2014). The impact of heat waves on children’s health: A systematic review. International Journal of Biometeorology, 58(2), 239–247. 10.1007/s00484-013-0655-x 23525899

[gh270219-bib-0087] Yang, X. , Xu, X. , Wang, Y. , Yang, J. , & Wu, X. (2024). Heat exposure impacts on urban health: A meta‐analysis. Science of the Total Environment, 947, 174650. 10.1016/j.scitotenv.2024.174650 38986701

[gh270219-bib-0088] Yoon, L. , Richardson, G. R. A. , & Gorman, M. (2024). Reflections on a century of extreme heat event‐related mortality reporting in Canada. GeoHealth, 8(2), e2023GH000895. 10.1029/2023GH000895 PMC10870068 38371353

[gh270219-bib-0089] Yu, J. , Moise, A. F. , Sahany, S. , Prasanna, V. , Chua, X. R. , Chen, C. , et al. (2025). High‐resolution dynamically downscaled projections of future extreme temperatures, heatwaves and exposure in Southeast Asia. Science of the Total Environment, 980, 179501. 10.1016/j.scitotenv.2025.179501 40306085

[gh270219-bib-0090] Yüzen, D. , Graf, I. , Tallarek, A.‐C. , Hollwitz, B. , Wiessner, C. , Schleussner, E. , et al. (2023). Increased late preterm birth risk and altered uterine blood flow upon exposure to heat stress. EBioMedicine, 93, 104651. 10.1016/j.ebiom.2023.104651 PMC10363435 37355458

[gh270219-bib-0091] Zaerpour, M. (2026). Projected exposure of Canadian children to rare, high‐severity heatwaves under continued warming. Zenodo. https://zenodo.org/records/21209856

[gh270219-bib-0092] Zaerpour, M. , Papalexiou, S. M. , & Pietroniro, A. (2025). Increasing tree canopy lowers urban air temperature by up to 1.5°C in heat‐prone areas. Npj Urban Sustainability, 5(1), 92. 10.1038/s42949-025-00277-x

[gh270219-bib-0093] Zaerpour, M. , Papalexiou, S. M. , & Tang, G. (2026). Widespread shift toward extreme dominated precipitation with pronounced trends in arid and Mediterranean regions. Scientific Reports, 16(1), 18415. 10.1038/s41598-026-47708-y PMC13265802 42009781

[gh270219-bib-0094] Zhang, Z. , Heerspink, H. J. L. , Chertow, G. M. , Correa‐Rotter, R. , Gasparrini, A. , Jongs, N. , et al. (2024). Ambient heat exposure and kidney function in patients with chronic kidney disease: A post‐hoc analysis of the DAPA‐CKD trial. The Lancet Planetary Health, 8(4), e225–e233. 10.1016/S2542-5196(24)00026-3 38580424

[gh270219-bib-0095] Zou, J. , Zhong, G. , Wang, L. L. , Katal, A. , Gaur, A. , Yan, S. , et al. (2026). Urban heatwaves and mortality: A socioeconomic and environmental study using a novel building heat vulnerability index. Cities, 169, 106558. 10.1016/j.cities.2025.106558

