# Supplementary material for: Projected Exposure of Canadian Children to Rare, High‐Severity Heatwaves Under Continued Warming
Source: Geohealth. 2026 Oct 26;10(10):e2026GH001873. doi: 10.1029/2026GH001873 (PMC13701080; doi:10.1029/2026GH001873)
Supplement: Supplementary file 1 [file GH2-10-e2026GH001873-s001.docx]

*Geohealth*

Supporting Information for

**Projected Exposure of Canadian Children to Rare, High-Severity Heatwaves Under Continued Warming**

Masoud Zaerpour^1,*^, Saeid Taleghanidoozdoozan^2^, Brian Jacob Steele^3^, Shadi Hatami^1^, Lincoln Linlin Xu^2^, Simon Michael Papalexiou^1,4^, Daniel Helldén^5^

1. Department of Civil Engineering, Schulich School of Engineering, University of Calgary, 2500 University Drive NW, Calgary, T2N1N4, Alberta, Canada
2. Department of Geomatics Engineering, Schulich School of Engineering, University of Calgary, 2500 University Drive NW, Calgary, T2N1N4, Alberta, Canada
3. Centre for Health Informatics, Community Health Sciences, Cumming School of Medicine, University of Calgary, 2500 University Drive NW, Calgary, T2N1N4, Alberta, Canada
4. Institute for Global Water Security, Hamburg University of Technology, 21073 Hamburg, Germany
5. Institutes for Transdisciplinary Scholarships, University of Calgary, 2500 University Drive NW, Calgary, T2N1N4, Alberta, Canada

* Corresponding author: Masoud Zaerpour ([masoud.zaerpour@ucalgary.ca](mailto:masoud.zaerpour@ucalgary.ca))

**Contents of this file**

Text S1: Expanded methods

**S1. Expanded methods**

# S1.1. Downscaling and bias-correcting

To characterize future climate conditions, we used bias-corrected daily $T_{\max}$ projections from CMIP6 models as provided by the Canadian Downscaled Climate Scenarios (CanDCS6) archive maintained by Environment and Climate Change Canada. CanDCS6 offers daily temperature fields at ~10 km resolution that have been statistically downscaled and bias-corrected to align with observed climate statistics. The downscaling uses the BCCAQv2 method—a quantile-mapping framework designed to preserve the structure of climate-change signals—and, in the multivariate products, a multivariate bias-correction approach (MBCn)(Cannon et al., 2015; Werner & Cannon, 2016). Both methods are implemented in the R package ClimDown(Hiebert et al., 2018). We extracted daily $T_{\max}$ from 14 available CMIP6 models and scenarios in CanDCS6, including low-emissions (SSP1-2.6), intermediate (SSP2-4.5), and high-emissions (SSP5-8.5) pathways. As with Daymet, the model fields were aggregated to the DA scale by averaging all grid cells overlapping each DA, yielding DA-level daily $T_{\max}$ time series for 57,932 DAs across Canada from 1981 to 2100. These DA time series form the basis for computing annual heatwave indices under each model and scenario and for classifying each DA into global-warming categories based on its simulated temperature change.

We leveraged children population data from the 2021 Canadian Census at the Dissemination Area (DA) level. DAs are the smallest standard geographic units used in Canadian census data, each comprising one or more neighboring blocks and typically containing 400–700 people(Government of Canada, 2021). For each DA, we extracted total population counts and age distributions (by single year or age group), enabling identification of the number of children under 5 years old and their corresponding birth cohorts. Our analysis focuses on both recent and future birth cohorts and the broader “childhood” population (ages 0–18). To project future distributions of children at the DA level, we utilized provincial-scale population forecasts from the Government of Canada and spatially downscaled them by assuming that future DA-level distributions follow the 2021 census age structure. By combining these demographic data with our climate projections, we quantified not only the geographic extent of extreme heat exposure, but also the population-level burden—specifically, how many children in each location are likely to be affected. All spatial datasets (Daymet grid, climate model outputs, and census polygons) were aligned to a common geographic framework, allowing consistent extraction of temperature time series for each DA.

Demographic information was drawn from the 2021 Canadian Census at the DA level. DAs are designed to be relatively homogeneous in population size and cover the entire country and typically containing 400–700 people(Government of Canada, 2021), making them well suited for spatially explicit exposure analysis. For each DA, we obtained the total population and the age distribution, reported either by single year or by age group. From these distributions we identified the number of children under 5 years of age living in each DA in 2021 and assigned them to birth-year cohorts. Our analysis focuses both on these recent and future birth cohorts and on the broader “childhood” period, defined as ages 0–18. To project the spatial distribution of children into the future, we used province-level child population forecasts from Statistics Canada and downscaled them to the DA scale under a simple but transparent assumption: the spatial pattern of child population across DAs within each province remains proportional to the pattern observed in the 2021 census. Combining these DA-level child counts with the DA-level climate projections allows us to estimate not only where extreme heat will intensify, but also how many children in each location are likely to be exposed. All spatial datasets (Daymet grid, CanDCS6 climate grids, and census polygons) were brought into a common geographic reference system so that temperature time series could be consistently extracted for each DA and directly linked to its resident population.

# S1.2. Calculation of HWMId

We used the Heat Wave Magnitude Index daily (HWMId) to quantify the severity of heatwaves at the DA scale. HWMId is designed to capture both the intensity and duration of heatwaves as a single annual index and is constructed relative to each location’s own climate(Grant et al., 2025; Russo et al., 2014, 2015). The computation proceeds in several steps. First, for each DA we defined a baseline climatology for $T_{\max}$ using a 30-year reference period. For each calendar day, we calculated a local daily threshold as the 90th percentile of $T_{\max}$ using a centered 31-day moving window over the baseline years. This yields a smooth sequence of thresholds $T_{\mathrm{thr}}(i)$that reflects the typical upper tail of the seasonal temperature cycle at that location.

A heatwave event at a given DA is then defined as any period of at least three consecutive days during which daily $T_{\max}$ exceeds the corresponding threshold $T_{\mathrm{thr}}(i)$. For every day $i$ within each identified heatwave, we compute a daily magnitude $M_{d}(i)$ that measures how extreme that day is relative to the distribution of historical annual maxima. Let $T_{(30y,25\%)}$and $T_{(30y,75\%)}$denote the 25th and 75th percentiles of annual maximum $T_{\max}$ over the same 30-year reference period. The daily magnitude is then given by

$$M_{d}\left( i \right)=\frac{T_{max}\left( i \right)-T_{thr}(i)}{T_{30y,75\%}-T_{30y,25\%}}$$

(1)

In essence, $M_{d}(i)$ measures how far above the threshold the temperature is, scaled by the historic variability of extremes, enabling meaningful comparison across different climates. For each contiguous heatwave of length $L$days, we define the event magnitude as the sum of daily magnitudes across the event given by:

$$M_{\text{event}}=\sum_{i=1}^{L} M_{d}(i)$$

(2)

and we define the annual HWMId for that DA and year as the maximum event magnitude among all heatwaves occurring in that year. Larger HWMId values correspond to heatwaves that are more intense, longer, or both.

We applied this procedure to both the historical Daymet-derived DA series (1981–2024) and the CanDCS6 DA series (1981–2100) for each CMIP6 model and SSP scenario. The result is an annual HWMId time series for every DA and model realization, forming the basis for both the historical mortality analysis and all future exposure calculations.

To distinguish severe events from more common heatwaves, we derived lifetime-scale threshold from the annual HWMId distributions. For the national-scale exposure analysis, we defined a Lifetime-Scale Heatwave Exposure (LHE) threshold for each DA as the 98th percentile of its annual HWMId values over the full simulation period. Exceedance of this threshold identifies years in which heatwaves are rare and unusually intense from the perspective of an individual’s lifetime at that location. Counting how often annual HWMId exceeds this LHE threshold provides a measure of how many “high-severity heatwave years” an individual is likely to experience.

To organize exposure results by global warming level, we classified each DA–model–scenario combination into warming categories based on its simulated change in mean annual temperature. We did not directly use global mean temperature from the CMIP6 models; instead, we treated the local annual temperature anomaly as a location-specific proxy for the level of global warming experienced in that DA.

For each CanDCS6 realization, we first computed the mean annual temperature at each DA over the baseline period. We then computed the mean annual temperature over the last 30 years available in that future projection (i.e., 2071–2100), representing the late-century climate state under that model and scenario. The difference between these two 30-year averages defines a local warming anomaly, for each DA. This ΔT represents how much warmer the late-century climate is relative to the late-20th-century baseline.

Each DA–model–scenario combination was then assigned to one of five simplified warming bins according to its ΔT: 1–3 °C (labeled “1 °C” for brevity); 3–5 °C (“3 °C”); 5–7 °C (“5 °C”); and 7 °C or greater (“7+ °C”). These bins represent ranges of projected local warming experienced at each DA and are used to compare exposure outcomes across locations experiencing different magnitudes of climate change. The trajectory classification was saved alongside each DA’s baseline mean, future mean, and ΔT, and all subsequent exposure metrics were stratified by these local warming trajectories. This allows us to interpret results in terms of worlds that have warmed by approximately 1 °C, 3 °C, 5 °C, or 7+ °C degrees relative to 1980–2010, while still reflecting the spatially heterogeneous nature of local warming.

For each contiguous heatwave event of length $L$ days, we then compute the event magnitude as the sum of the daily magnitudes over those $L$ days: $M_{\text{event}}=\sum_{i=1}^{L} M_{d}(i)$. The HWMId value for a given year is defined as the *maximum* event magnitude observed in that year(Wang & Yan, 2021). HWMId is a dimensionless index, with larger values indicating more severe and intense heatwaves. For example, an HWMId above 15 corresponds to an extremely severe heatwave event(Zittis et al., 2021). This metric has the advantage of being comparable across different climates and time periods, since it accounts for local climate norms and provides a standardized measure of heatwave magnitude. We computed HWMId for each year from 1981 through 2100 for every DA in our dataset. The calculation was done separately for each climate model simulation in the ensemble. The result is a time series of annual HWMId values for each DA under each scenario, which captures how heatwave severity evolves over time at the local level.

# S1.3. Childhood and Lifetime Exposure to High-Severity Heatwaves

Finally, we combined the DA-level HWMId time series, the LHE threshold, and the age-structured population data to quantify children’s exposure to severe heatwaves under different warming trajectories. We defined childhood as the period from birth through age 18. For a child born in year $y$ in a given DA, their childhood exposure trajectory is the sequence of annual HWMId values from year $y$through year $y+18$. Using the DA-level HWMId time series from CanDCS6, we constructed such trajectories for a range of birth cohorts spanning the current and future periods the 21st century.

For each cohort and DA, we then counted how many childhood years had annual HWMId exceeding the local LHE threshold. This count represents the number of years in which that child experiences a high-severity heatwave, in the sense of exceeding a rare, lifetime-scale benchmark. Because each DA–model–scenario combination is also assigned to one of the ΔT warming bins described above, these exposure counts can be summarized separately for regions projected to warm by about 1–3 °C, 3–5 °C, 5–7 °C, or ≥7 °C. In this way, we can answer questions such as: for children born in 2030 or 2040, how many LHE-level heatwave years are expected during childhood in areas on a ~1 °C warming trajectory compared to areas on a ~5 °C or 7+ °C trajectory?

To move from per-child trajectories to population-level exposure, we weighted these counts by the number of children living in each DA. The 2021 census provides DA-level counts of children aged 0–4; we treated these as representative of near-term birth cohorts. For future decades, we used province-level child population projections and distributed them across DAs in proportion to the 2021 age distribution, thereby preserving the spatial pattern of where children reside within each province. Combining these population counts with the modeled number of LHE-exceedance years per cohort and warming category allowed us to estimate both the proportion of children who will experience at least one LHE-level heatwave during their lifetime and the total number of such events that different cohorts are likely to face under alternative warming pathways.

**References**

Cannon, A. J., Sobie, S. R., & Murdock, T. Q. (2015). *Bias Correction of GCM Precipitation by Quantile Mapping: How Well Do Methods Preserve Changes in Quantiles and Extremes?* https://doi.org/10.1175/JCLI-D-14-00754.1

Government of Canada, S. C. (2021, November 17). *Dictionary, Census of Population, 2021 – Dissemination area (DA)*. https://www12.statcan.gc.ca/census-recensement/2021/ref/dict/az/definition-eng.cfm?ID=geo021

Grant, L., Vanderkelen, I., Gudmundsson, L., Fischer, E., Seneviratne, S. I., & Thiery, W. (2025). Global emergence of unprecedented lifetime exposure to climate extremes. *Nature*, *641*(8062), 374–379. https://doi.org/10.1038/s41586-025-08907-1

Hiebert, J., Cannon, A. J., Murdock, T., Sobie, S., & Werner, A. (2018). ClimDown: Climate Downscaling in R. *Journal of Open Source Software*, *3*(22), 360. https://doi.org/10.21105/joss.00360

Russo, S., Dosio, A., Graversen, R. G., Sillmann, J., Carrao, H., Dunbar, M. B., Singleton, A., Montagna, P., Barbola, P., & Vogt, J. V. (2014). Magnitude of extreme heat waves in present climate and their projection in a warming world. *Journal of Geophysical Research: Atmospheres*, *119*(22), 12,500-12,512. https://doi.org/10.1002/2014JD022098

Russo, S., Sillmann, J., & Fischer, E. M. (2015). Top ten European heatwaves since 1950 and their occurrence in the coming decades. *Environmental Research Letters*, *10*(12), 124003. https://doi.org/10.1088/1748-9326/10/12/124003

Wang, J., & Yan, Z. (2021). Rapid rises in the magnitude and risk of extreme regional heat wave events in China. *Weather and Climate Extremes*, *34*, 100379. https://doi.org/10.1016/j.wace.2021.100379

Werner, A. T., & Cannon, A. J. (2016). Hydrologic extremes – an intercomparison of multiple gridded statistical downscaling methods. *Hydrology and Earth System Sciences*, *20*(4), 1483–1508. https://doi.org/10.5194/hess-20-1483-2016

Zittis, G., Hadjinicolaou, P., Almazroui, M., Bucchignani, E., Driouech, F., El Rhaz, K., Kurnaz, L., Nikulin, G., Ntoumos, A., Ozturk, T., Proestos, Y., Stenchikov, G., Zaaboul, R., & Lelieveld, J. (2021). Business-as-usual will lead to super and ultra-extreme heatwaves in the Middle East and North Africa. *Npj Climate and Atmospheric Science*, *4*(1), 20. https://doi.org/10.1038/s41612-021-00178-7
